# Effect of Surface Textures and Fabrication Methods on Shear Bond Strength Between Titanium Framework and Auto-Polymerizing Acrylic Repair Resin

**DOI:** 10.7759/cureus.48883

**Published:** 2023-11-16

**Authors:** Ghada O Alrabeah

**Affiliations:** 1 Department of Prosthetic Dental Sciences, College of Dentistry, King Saud University, Riyadh, SAU

**Keywords:** milled titanium, autopolymerizing acrylic resin, air-abrasion, shear bond strength, denture repair

## Abstract

The aim of the study was to evaluate the effect of airborne particle abrasion (using different sizes of alumina particles) on the shear bond strength (SBS) between cast and milled titanium metal frameworks and auto-polymerizing acrylic repair resin. Forty flat cylindrical titanium disks were divided into two main divisions: cast and milled titanium. The two divisions were further subdivided into four groups based on metal surface treatment. Three particle sizes of aluminum oxide air abrasive powders (50µm, 110µm, and 250µm) were used for metal surface treatment by airborne particle abrasion. One group was the control group with no surface treatment. Auto-polymerizing acrylic repair resin was applied to all titanium disks. The specimens were subjected to SBS testing using a universal testing machine (Instron Corporation, Norwood, Massachusetts, United States). Surface evaluation was performed using a scanning electron microscope. One-way ANOVA was used for statistical analysis. The results showed a significant increase in SBS after airborne particle abrasion of both milled and cast titanium groups (p<0.001). The SBS was directly proportional to the size of the aluminum oxide particles. The milled titanium group showed higher SBS values than the cast group when the surface was not treated with alumina particles (p < 0.001) and when the surface was treated with the smaller particle sizes of 50 µm, whereas the cast group demonstrated higher SBS values than the milled group (p < 0.01) when the particle size was increased to 110 µm and 250 µm. It could be concluded that SBS between titanium metal frameworks and auto-polymerizing repair acrylic resin was directly related to the size of the alumina airborne particle abrasives. The fabrication method of the titanium framework also influenced the SBS as the untreated milled frameworks demonstrated favorable SBS values compared to the untreated cast frameworks.

## Introduction

Removable partial dentures (RPDs) still prevail as an effective and economical treatment option for partially edentulous patients to replace their missing teeth and improve their life quality [1]. These prostheses are made of polymethylmethacrylate (PMMA) denture acrylic resin overlaying a metal base framework [2]. Base metal alloys such as cobalt-chromium (Co-Cr) alloys are widely used over gold alloys for RPDs and provide a rigid and inexpensive denture metal base. However, the allergic and toxic potentials of some elements in these alloys to some patients have been of concern [3]. Due to its outstanding biocompatibility, superior corrosion-resistant properties, and light weight owing to its low density, titanium (Ti) has been increasingly used in clinical practice for removable prostheses [3,4]. Ti frameworks are cast using the lost-wax technique and remain functional for the long term without catastrophic failure [5,6]. With the introduction of computer-aided design/computer-aided manufacturing (CAD/CAM) technology, the fabrication of milled Ti frameworks became popular as it overcomes some of the problems encountered with metal casting such as distortion and porosity due to expansion and contraction that occur during casting procedures [5-7].

One shortcoming of RPDs is the susceptibility of the overlaying acrylic resin to fracture at the metal-resin interface when dropped or overloaded beyond its fracture strength [2,8,9]. Repair of the fractured acrylic provides a feasible and economic solution over the construction of a new replacement RPD [8,9]. Denture repair involves different materials and techniques such as visible light-polymerized, microwave-polymerized, auto-polymerized (self-cure), and heat-polymerized acrylic resins [8]. The use of auto-polymerized, chemically activated resins allows a rapid repair procedure because the step of denture flasking is omitted; hence, no heat is present to release stresses within the acrylic resin, which yields a repaired denture with enhanced accuracy [10]. However, the resin-to-metal bond is a key factor for the success of the repaired RPD. The adhesion of repair resin to metal frameworks is influenced by the metal surface treatments achieved by different mechanical and chemical methods [3,9].

Various methods have been implemented to improve the bonding of acrylic resins to metal framework substructures including electrolytic etching, chemical etching, silica coating, spark erosion, laser application, and the application of metal primers [3]. Mechanical retention is achieved through macromechanical means such as mesh, beads, and posts and through micromechanical means such as air abrasion, acid, and electrolytic etching [9]. The technique of airborne particle abrasion using aluminum oxide particles has been commonly used for improving the resin-metal bond [3]. Air abrasion produces a rough surface, which increases the surface area for resin attachment. Alumina particles used for air abrasion in dental laboratory procedures are available in various sizes ranging from 25 µm to more than 250 µm. Such variation in grain size is assumed to produce different surface textures that could provide different mechanical interlocking patterns between repair resin and metal substructure. However, the effect of air abrasion with alumina particles on the metal surface is influenced by several factors, including the pressure applied during air abrasion and the metal surface hardness, which is material-dependent [11,12]. The surface hardness influences the penetration depth of alumina particles into the metal surface. Pure Ti is known to have low Vickers hardness ranging from 130 HV to 210 HV [5,11,12]. Ti hardness is also influenced by its fabrication method. Wang et al. demonstrated that cast Ti exhibited higher hardness values than milled Ti, 181.7 HV and 152 HV, respectively [12].

Based on the findings of previous studies, and considering the abundance of digital milling fabrication techniques for RPD Ti frameworks nowadays, it is necessary to investigate the effect of sandblasting techniques using different alumina particle sizes as a means for micromechanical retention between the repair resin and Ti metal frameworks fabricated by traditional casting and recent digital milling. Therefore, the aim of the present study was to evaluate the effect of metal surface textures created by different sizes of alumina particles on the shear bond strength (SBS) between cast and milled Ti metal frameworks and auto-polymerized acrylic repair resin.

## Materials and methods

The study was conducted in the physical laboratory of King Saud University Hospital and College of Dentistry Research Center, Riyadh, Saudi Arabia, in accordance with the Declaration of Helsinki. The study was approved by the Institutional Review Board (Ethics Committee) of King Saud University, College of Dentistry Research Center (project no. FR 0681, June 11, 2023).

Fabrication of test specimens

**Figure 1:**
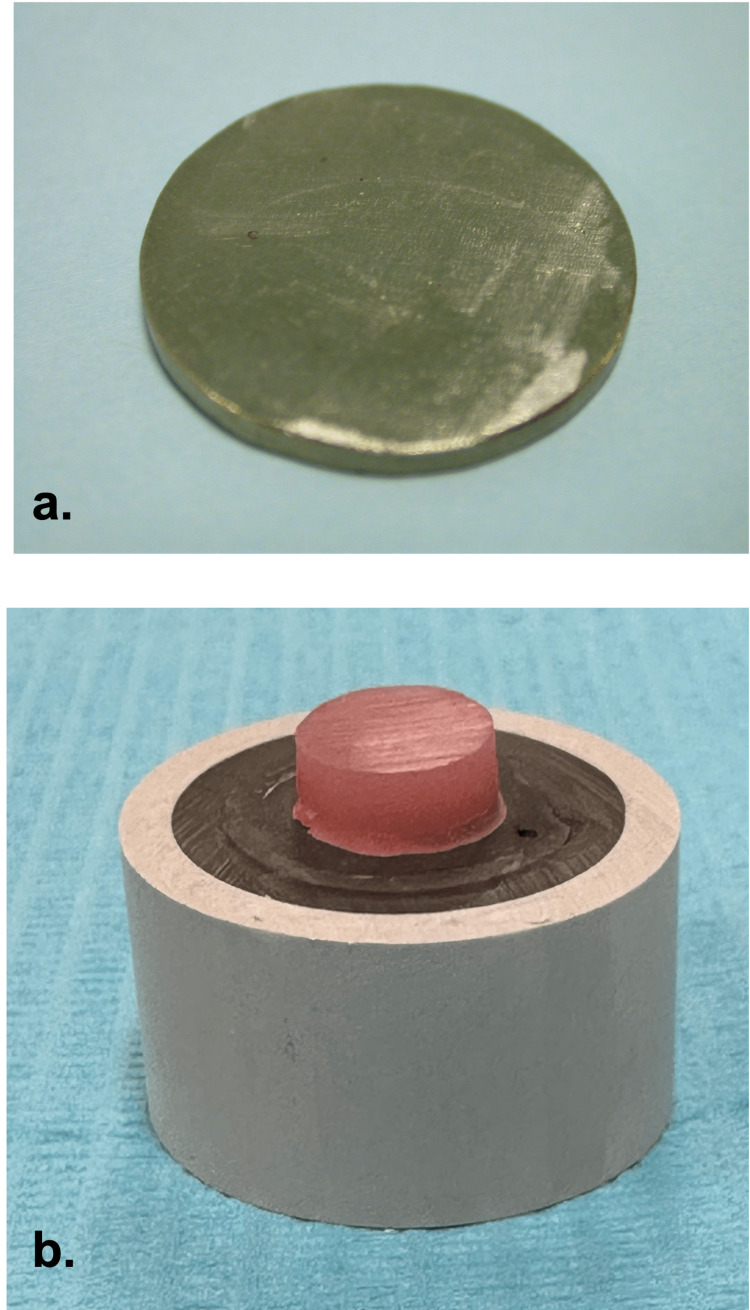
(a) Titanium disk; (b) Auto-polymerized acrylic repair resin applied over embedded titanium disk representing the final form of the specimens

The specimens used in this investigation were in the form of flat cylindrical Ti metal disks (Figure 1a), over which self-cure denture acrylic material was applied (Figure 1b). 

The Ti metal substructures were constructed by two different methods resulting in two main groups: milled Ti specimens (M) and cast Ti specimens (C). Each group consisted of 20 specimens. Milled Ti specimens were prepared by sectioning the as-received machined cylindrical grade 1 commercially pure Ti (CPTi) ingots [5] (Tritan, Dentaurum GmbH & Co. KG, Ispringen, Germany) to dimensions of 20 mm in diameter and 2 mm in thickness using a slow-speed, water-cooled abrasive cut-off wheel (Universal Centre Lathe SN50 Classic, TRENS SK, Inc., Trenčín-Kubrá, Slovakia). 

Cast Ti specimens were fabricated to the same dimensions as the milled group using the lost-wax technique. Molten inlay wax was poured into a specially constructed mold with the same configuration as the milled group specimens. The wax disks were converted into CPTi specimens following the lost wax technique steps of spring, investing, and then casting following the manufacturer's recommendations. 

Metal surface preparation

Ti disks from each group underwent finishing procedures using tungsten carbide cone bur and Suva rubber diamond bur (SUVA Lapidary, LLC, Kokomo, Indiana, United States), following manufacturer recommendations. Finishing was performed in one direction to avoid trapping particles in folds of metal, and local overheating was avoided by following the manufacturer's recommendation for CPTi to not exceed 10,000 rpm during finishing. After finishing, all metal disks were steam cleaned (Triton SLA steam cleaner, Bego Corporation du Canada Inc, Quebec City, Canada) and wiped with alcohol.

The Ti specimens were embedded in clear auto-polymerizing resin (Meliodent, Kulzer GmbH, Hanau, Germany) within plastic rings, leaving one surface exposed for later surface treatment and auto-polymerising acrylic resin application. 

The total sample size was 40 with 20 samples divided into two divisions, milled and cast. Each main Ti division (n=20) was randomly divided into four sub-groups based on metal surface treatment with five samples in each group. One group was the control with no further surface treatment after finishing, and the remaining three groups were airborne particle abraded. Three particle sizes of aluminum oxide air abrasive powders (50µm, 110µm, and 250µm) (Cobra Abrasives, Renfert GmbH Company, Hilzingen, Germany) were used for further metal surface preparation by airborne particle abrasion. The finished group that did not receive air abrasion represented the control group (0). Ti specimens were airborne particles abraded (Duostar; BEGO Bremer Goldschlägerei Wilh. Herbst GmbH & Co. KG, Bremen, Germany) with their corresponding aluminum oxide powder as shown in Table 1 at a constant pressure of 3 bar. The distance between the tip of the nozzle and the surface of each specimen was 15 mm with approximately a 45º jet angle. Each specimen was abraded in seven runs at a rate of one run every two seconds. After airborne particle abrasion, all Ti specimens were steam cleaned. The resultant eight groups (n=5) were coded according to surface treatment and metal fabrication methods as shown in Table 1.

**Table 1:** Codes of specimens relative to surface treatment and metal fabrication method

Surface Treatment	Metal Fabrication Method	Code
Finishing only	Milled	M0
Finishing only	Cast	C0
Airborn particle abrasion 50 µm	Milled	M50
Airborn particle abrasion 50 µm	Cast	C50
Airborn particle abrasion 110 µm	Milled	M110
Airborn particle abrasion 110 µm	Cast	C110
Airborn particle abrasion 250 µm	Milled	M250
Airborn particle abrasion 250 µm	Cast	C250

Application of auto-polymerizing acrylic repair resin

To provide a uniform bonding area, masking tape with a 10 mm diameter hole was placed on the exposed surface of the embedded Ti disks. A custom-made putty mold with a 10 mm internal diameter and 4 mm thickness was positioned over the hole for acrylic packing. The exposed surfaces of the disks were painted with a monomer bonding agent and were allowed to dry for two minutes as instructed by the manufacturer. Self-cure denture acrylic resin (Meliodent Rapid Repair, Kulzer GmbH) was mixed in the ratio of 10 g powder: 7 mL liquid according to the manufacturer’s instructions, and carefully hand-packed into the putty mold to minimize air bubble entrapment. After polymerization, the putty mold and masking tapes were removed. All the finished specimens were then stored wet at 37°C for 24 hours before testing. Materials used in the study are presented in Table 2.

**Table 2:** Materials used in the study

Material	Composition	Name/Brand and Manufacturer
Commercially pure titanium	Ti >99%, N<0.05%, Fe<0.5%, O<0.4%, C<0.1%, H<0.15	Tritan pure titanium; Dentaurum GmbH & Co. KG, Ispringen, Germany
Sandblasting particles	99.6% Al_2_O_3_	Cobra abrasives; Renfert GmbH Company, Hilzingen, Germany
Repair acrylic	Powder: polymethyl-methacrylate Liquid: methyl-methacrylate	Meliodent Rapid Repair; Kulzer GmbH, Hanau, Germany

SBS testing

**Figure 2:**
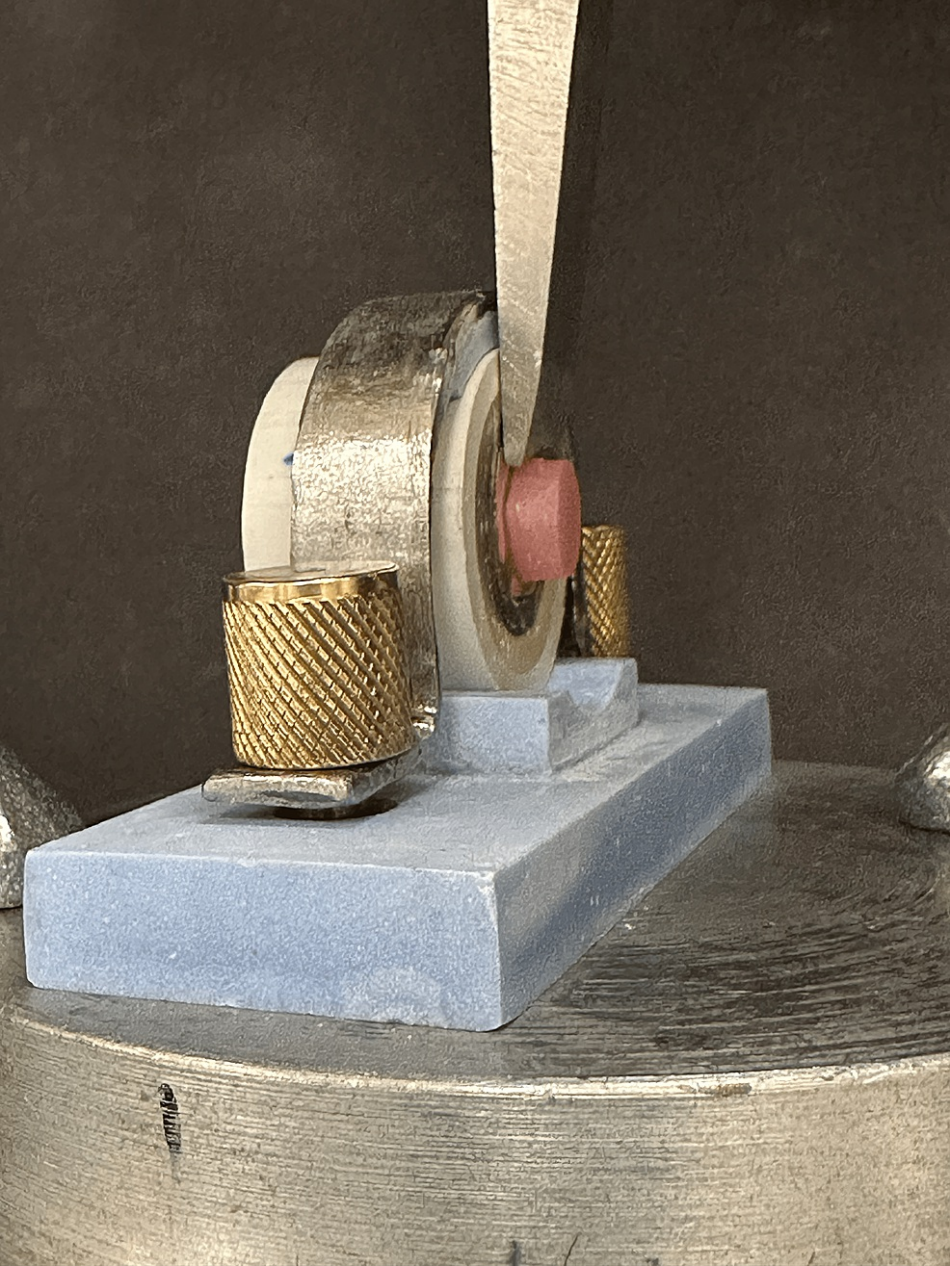
Mounted specimen on the universal testing machine for SBS SBS: shear bond strength

After the specimens were dried, the SBS for all groups was measured. Acrylic-to-metal bond strength was evaluated using a universal testing machine (Instron 5965; Instron Corporation, Norwood, Massachusetts, United States) at a 1 mm/min crosshead speed (Figure 2). Testing proceeded for either one minute or until failure. The SBS (in MPa) was calculated by dividing the maximum load (N) by the cross-sectional area (mm^2^) for each denture self-cure acrylic resin over a Ti substrate.

Statistical analysis

SBS values were analyzed using IBM SPSS Statistics for Windows, Version 26.0 (Released 2019; IBM Corp., Armonk, New York, United States). Descriptive statistics (mean and standard deviation) were used to describe the quantitative outcome variable SBS. The data was normally distributed. One-way analysis of variance (ANOVA) and Tukey's post-hoc tests for multiple comparisons were used in the statistical analysis to compare SBS mean values with confidence intervals of 95% for all samples to determine differences between surface treatment within metal fabrication method and independent sample T-test to determine differences between two fabrication methods within the same surface treatment. The significance threshold was set at α<0.05.

Surface evaluation of treated Ti disks

Additional metal disks (two representing each group) were fabricated following the same methods described earlier. These metal specimens were used for surface evaluation and did not receive acrylic. Surface roughness was evaluated using a profilometer (Surtronic 10; Taylor Hobson, Leicester, United Kingdom). To measure the roughness profile value in µm, the diamond stylus (5-µm tip radius) was moved across the metal surface under a constant load of 10 mN and a speed of 2 mm/s during testing. This procedure was repeated three times at a different location for each specimen to obtain its general surface characteristics. The average values of these measurements were the Ra values. Further analysis was then performed on the same specimens under a scanning electron microscope (SEM) (JSM-636 OLV; JEOL Ltd., Akishima, Tokyo, Japan). The SEM photomicrographs were made with x1000 magnification at a different region of each specimen for visual inspection.

## Results

SBS

The mean and standard deviation values of SBS of all eight study groups of fabrication method and surface treatment combinations are presented in Table 3. 

**Table 3:** Comparison of mean values of shear bond strength among the two fabrication methods and the four surface treatments. *P value was significant at P<0.05 for comparisons between the fabrication methods at each surface treatment. ** P value was significant at P<0.05 for comparisons between surface treatments at each fabrication method.

Surface Treatment	Milled	Cast	*Anova
	Mean (Sd.)	Mean (Sd.)	P value
0	0.29 (0.06)	0.11 (0.01)	< 0.001
50 µm	1.56 (0.10)	1.12 (0.25)	< 0.001
110 µm	1.88 (0.07)	2.03 (0.15)	< 0.01
250 µm	2.13 (0.11)	2.82 (0.18)	< 0.001
**Anova P value	< 0.001	< 0.001	

**Figure 3:**
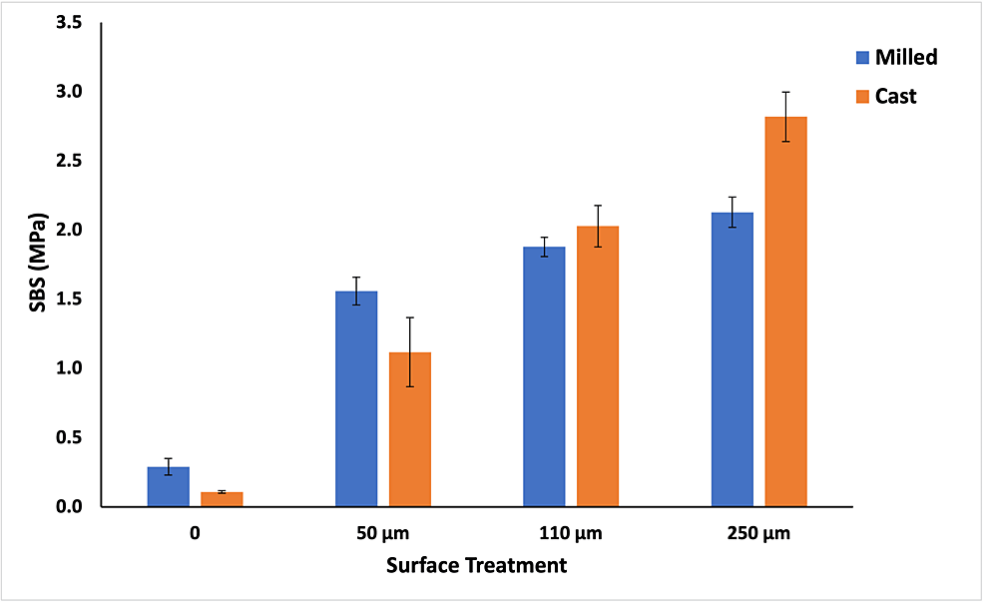
Comparison of mean values of SBS (MPa) between the milled and cast groups and four surface treatments (0.00, 50µm, 110µm, and 250µm) SBS: shear bond strength

Overall, there was a significant increase in shear bond strength after airborne particle abrasion of both milled and cast Ti groups (p<0.001) (Figure 3, Table 3). The larger the size of the aluminum oxide particles, the greater the SBS. The highest SBS was recorded for the cast Ti group treated with 250 µm (2.82 MPa) while the least SBS was observed in the untreated cast group (0.11 MPa). 

The SBS was also influenced by the fabrication method where the milled Ti group showed higher SBS values than the cast group when the surface was not treated with alumina particles (p < 0.001) and when the surface was treated with the smaller particle sizes of 50 µm (Table 3). When the particle size increased to 110 µm and 250 µm, the cast group demonstrated higher SBS values than the milled group (p < 0.01) (Table 3).

Surface evaluation of treated Ti disks

**Figure 4:**
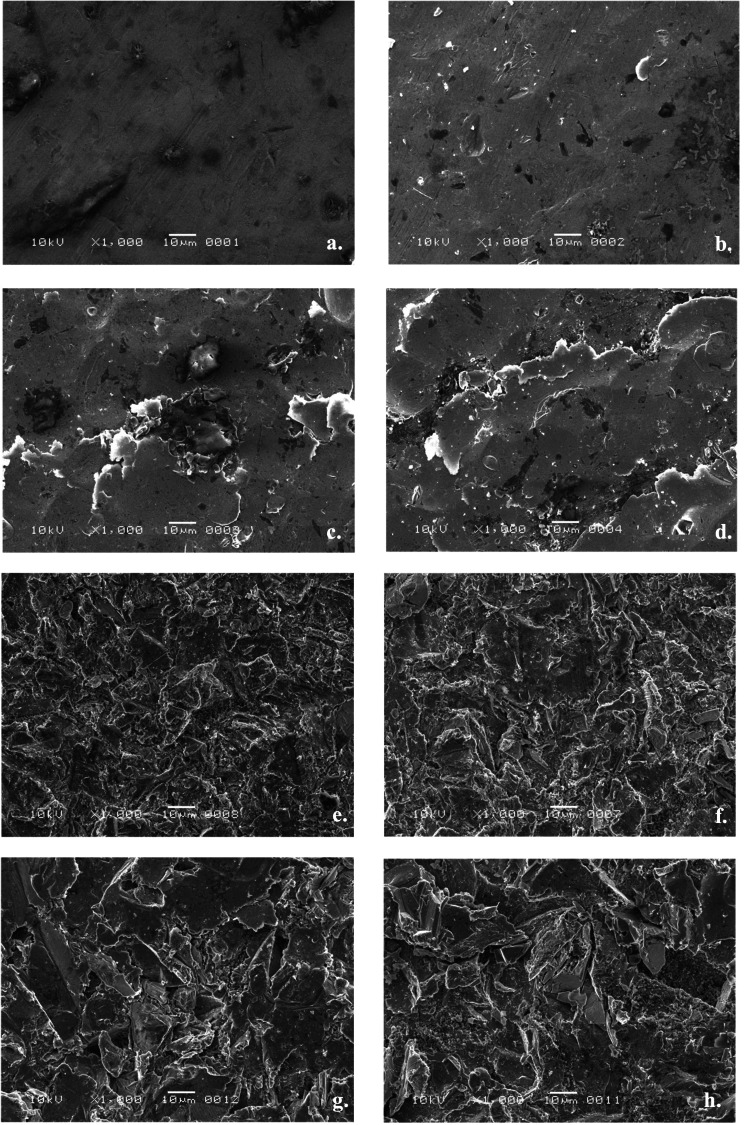
SEM evaluation SEM images of the cast and milled titanium disks with different surface textures at 1000x magnification: (a) C0; (b) M0; (c) C50µm; (d) M50µm; (e) C110µm, (f) M110µm; (g) C250µm; (h) M250µm SEM: scanning electron microscope

Surface topography and SEM interpretation indicated that surface roughness increased as the particle size of aluminium oxide-abrasive powder increased for both milled and cast Ti (Table 5, Figure 4). The cast and milled Ti surfaces treated by 250µm aluminum oxide particles were rougher and more abraded and had sharper edges than the 110µm and 50 µm treated surfaces (Figures 4g, 4h). The Ra values were influenced by the fabrication method where the milled group had a rougher surface when sandblasted with 50µm particle size (Ra=1.45µm) compared with the cast group (Ra=1.15µm). With 250µm aluminum oxide particles, the cast Ti group demonstrated a rougher surface (Ra=2.6µm) than the milled Ti group (Ra=2.15µm). 

**Table 4:** Mean Ra values (µm) of the additional metal disks after surface treatment with airborne particle abrasion.

Group Code	Ra Value (µm)		Average Ra Value (µm)
	Sample 1	Sample 2	
M50	1.5	1.4	1.45
M110	1.9	1.8	1.85
M250	2.1	2.2	2.15
C50	1.1	1.2	1.15
C110	1.9	1.8	1.85
C250	2.5	2.7	2.6

## Discussion

In real-world clinical settings, a frequent problem with RPDs is the detaching of the acrylic resin denture base from the metal framework [9]. Auto-polymerizing acrylic resin has usually been employed as a convenient solution in attempts to repair these broken denture bases, with varying degrees of success [8-10]. To predict the success of the repaired denture, it is critical to understand if the bond strength between the material of the repaired denture base and the metal framework is optimal or not [3,13]. 

The greatest force a material can withstand before failing under tension is known as shear strength [13]. SBS, which applies a shear pressure directly to the interface between repair material and denture metal base, has been widely used as one of the most standard and versatile bond strength tests [14]. Therefore, it is crucial to look at the SBS of material interfaces. Several mechanical and chemical surface treatments of the denture bases were tried to increase the shear bonding capacity between the fractured denture base and the repair material [15]. The bonding between the repairing denture base material and the metal denture frameworks has been improved using a variety of surface treatments and procedures [16]. The protocol of airborne particle abrasion, often described as sandblasting, with aluminum oxide powder particles has been accepted as an effective micromechanical technique for roughening the metal denture base surface and therefore increasing the surface area available for bonding the repair acrylic resin to the metal substructure [3,13,16]. Because it is simple, fast, and available in all laboratories, this surface treatment has been tested in the present study with a special focus on the effect of various alumina particle sizes for sandblasting as a mechanical way of improving the bonding between Ti denture framework and auto-polymerizing acrylic resin.

The SBS of the auto-polymerizing repair resin was significantly increased in the current study because the size of alumina particles for surface treatment was increased before denture base repair. In other words, the SBS was directly related to the alumina particle size; thus, the null hypothesis of no change in the SBS because of the change in the alumina partial size change for the sandblasting was rejected. The results are consistent with the work of Jaber et al., who demonstrated that aluminum oxide particles affected the bond strength of resins to base metal alloys and found that employing 110μm aluminum oxide particles significantly increased the roughness of the surface of the base metal alloy [17]. According to Jagger et al.'s research, the friction between the denture base and the repair material is further increased by a rough surface, necessitating a stronger debonding force at the interface [18].

Before repairing an acrylic denture foundation, Amarnath et al. suggested sandblasting it after discovering that the bond strength between auto-polymerized resin and the denture base was 2.90 MPa in the control group and 7.56 MPa in the sandblast group [19]. The maximum SBS value in the current study was 2.82 MPa recorded for the cast Ti group treated with 250μm, which is far below the value reported by Amarnath et al.; this is because the repair was between two acrylic resin materials, whereas in the current study, the repair was between dissimilar materials, acrylic resin and metal substructure, which lacked chemical adhesion [19]. Using 50μm aluminum oxide particles to treat the denture base surface, Minami et al. have also demonstrated enhanced bond strength between the repair resin and the denture base [20]. The authors declared that a larger size of aluminum oxide particles would further enhance the bond strength [13,20]. In an in vitro study, Kumar et al. tested the SBS of auto-polymerizing and light-cured acrylic repair resin materials to fractured denture bases using different surface treatment protocols [13]. Among the tested treatment protocols, one group was subjected to air abrasion with 50μm aluminum oxide_ _particles and another with 150μm aluminum oxide particles. Their results showed that air abrasion with alumina particles increased the SBS in the group repaired with light-cured repair resin only and that the increase was inversely related to particle size [13]. This finding contradicts the current findings, in which enlarging the particle size directly increased the bond strength. This could be explained by the earlier reports of Rudawska et al.. which claimed that the bond strength could decrease when the adherend surface is too rough [21], given that excess roughness minimizes the ability for adhesive penetration and leads to greater void formation, therefore extending the concentration of localized stress [21]. Such contradiction in the results could also be attributed to the fact that Kumar et al. combined the 50μm air abrasion regime with the application of acetone for 30 seconds, which enhanced the chemical adhesion of the light-cured repair resin [13]. That the present study used one type of repair resin is considered a limitation; therefore, the results should not be generalized for all repair materials.

The results are equivalent and even better when the findings of the current approach are compared with those of a study using an expensive technology such as the use of lasers for surface treatments [3]. Alumina sandblasting increases surface roughness while also enhancing bonding surface area and generating surface energy [21]. Additionally, it cleans the denture base material's surface of any impurities or dust as well as the oxide layer. All these aid in creating a chemical link between metal framework alloys and self-curing acrylic resin [21]. The outcomes of sandblasting with 50μm alumina particles were found to be the lowest of all the groups in the current investigation. PMMA particles, which make up most of the polymeric resin and are about 100μm in size, cannot completely penetrate surfaces that have been roughened with minuscule aluminum oxide particles. Alumina particles that cling to the metal surface also prevent PMMA particles from freely flowing across the rough surface and impair a solid connection [22]. These factors might help explain why our study found lower bond strength values for smaller aluminum oxide particles and greater bond strength values for larger aluminum oxide particles.

The majority of the earlier experiments tested the SBS between auto-polymerizing acrylic resin and non-precious metal alloys, such as Co-Cr alloys [23]. The utilization of the CPTi denture framework constructed through the employment of two distinct fabrication procedures was a critical focus of the present investigation. Traditionally, Ti metal frameworks, CPTi and Ti-alloy, were cast; however, laboratory shortcomings of cast Ti frameworks still exist, including long duration for Ti burnout, inferior castability, thick Ti oxide reaction layer on the surface, difficult polishing, and high initial costs [5].

The development of CAD and digital fabrication techniques has allowed the production of more accurate and homogeneous Ti prostheses in a shorter time [6,12,24]. Digital fabrication methods could be subtractive or additive techniques. Milling is a subtractive process that uses CAD/CAM machines to cut and shape the block of alloy or disk to the final framework according to the pre-designed model [12]. Additive manufacturing techniques, on the other hand, employ selective laser melting (SLM) as a three-dimensional (3D) printing technology to form a metal framework from metallic powders that are melted and fused using a high-power-density laser [12]. Although additive manufacturing overcomes some of the drawbacks of the milling process, including long processing time, difficulty in shaping complicated designs, and undercutting and wearing of cutting tools, which harms framework accuracy, it is not free of critical shortcomings [25]. A crucial limitation of this technology involves the appearance of a step-like structure due to the layering of the material, thus creating a rough surface. The evolution of a hybrid technique that combines repeated laser sintering and high-speed milling is appealing for mitigating both drawbacks [25,26]. The lack of an additive fabrication method in the current investigation presents another limitation.

Pure Ti fabricated by additive techniques was found to have the highest Vickers hardness (402.05 HV) compared with cast (181.7 HV) and milled samples (152.9 HV) [12]. This could be correlated with the results of the present study, which showed that the milled Ti samples that were untreated or treated with smaller particle sizes of 50μm had higher SBS than their correspondents from the cast group. The increased SBS value for the untreated milled disks and those treated with smaller particle sizes of 50μm compared to their counterparts from the cast group could also be attributed to the presence of a thick titanium oxide layer on the cast disks that was difficult to remove with the small 50μm aluminum oxide_ _particles. This thick metal oxide layer negatively affects the bond strength of the overlying denture base material [5]. Although it seems reasonable to infer that the softer the metal, the rougher the air-abraded surface texture, this relationship was not seen between the milled and cast groups when using larger particle sizes, which did not differ in Ra values at 110μm. In fact, when 250μm particles were used, the Ra values for the cast Ti were higher than those of the presumably softer milled samples. Additionally, the airborne particle-abraded Ti surfaces did not show an obvious difference between the cast and milled Ti groups in the SEM images within the same aluminum oxide particle size, irrespective of the difference in their Ra and SBS values. This was in line with the earlier observations of Kawaguchi et al., who presented no differences in the SEM images between the metal surfaces of the tested Ti and CoCr alloys subjected to sandblasting with 50μm aluminum oxide particles regardless of the difference in hardness and SBS. The authors claimed that such observation highlights the necessity of chemical adhesion as a bonding method for a successful resin-to-metal bond [2]. 

The three airborne particle abrasion treatments used in this study resulted in different surface textures judged by surface analysis using a profilometer and observations under the scanning electron microscope. The appearance of the micromechanical roughened textures observed among the three particle sizes correlated with the surface roughness values (Ra) within each fabrication method. This was in line with observations by Nergiz et al., who demonstrated greater surface roughness of Ti after abrasion with 110μm particle size than that with 50μm particle size [27]. Yanagida et al., on the other hand, found no correlation between the roughness values and aluminum oxide particle sizes of 50μm and 110μm although the 110μm air abraded Ti samples appeared rougher under microscopic examinations. The authors attributed their findings to the remaining embedded alumina with the Ti matrix, therefore influencing the roughness values for both particle size treatments [28].

It was demonstrated in an earlier study that the bond strength was greater as the bonding surface became rougher [29]. This finding supports the results of the present study, as there was a correlation between the degree of roughness (Ra) by airborne particle abrasion and the SBS. Currently, several studies have investigated the employment of chemical agents such as an adhesive primer to strengthen the contact between adhesive resins and metal frameworks [3,13,16,30]. According to Yoshida et al.'s research, the interaction between the functional monomers of adhesive primers and the oxide layer produced on the metal surface strengthens the connection between the metal and acrylic resin [30]. This technique creates a strong chemical link between the resin and the metal alloys by causing the oxide layer that forms on the surface of basic metal alloys to bond chemically with the monomer. In earlier investigations, sandblasting with aluminum oxide particles followed by the application of a metal primer increased the binding strength between the metal and the acrylic resin compared with samples on which the metal primer was not applied [23,28]. Moreover, airborne particle abrasion of the metal surface with alumina particles generates a contact surface area that is appropriate for both micromechanical and chemical attachment; therefore, it was suggested that air abrasion using alumina must be implemented as a mandatory pretreatment step for chemical adhesion [28]. Accordingly, in the current work, the monomer was used to increase the bond strength after the specimens were sandblasted but before the auto-polymerizing repair was applied. Sandblasting and applying the monomer as a chemical agent are both simple procedures that may be done in any laboratory setting.

The use of one brand of auto-polymerized acrylic resin and monomer is a limitation of the current investigation. The absence of an oral environment simulation around the test specimens is another restriction that should be considered in the future. The oral cavity temperature, pH fluctuations, and dynamic fatigue loads that could significantly affect the outcomes were not assessed in this study. Further research involving the emerging hybrid manufacturing process that combines additive and subtractive digital techniques must be considered to determine the optimum techniques for strengthening the bond between the Ti framework and the self-cure acrylic denture foundation that ensures long-term clinical durability.

## Conclusions

Under the limitations of the present study, it could be concluded that following airborne particle abrasion of both milled and cast titanium denture base framework, there was a significant improvement in SBS with the auto-polymerizing acrylic denture base material. The SBS was directly related to the aluminum oxide particle sizes as it increased with the increase in the size of the aluminum oxide particle. The SBS was also influenced by the fabrication method where the milled titanium group showed higher SBS values than the cast group when using smaller particle size (50µm) while higher SBS values were observed in the cast group when using larger particle sizes (110µm and 250µm).
